# Cardio-Metabolic Risk Factors in Scottish South Asian and Caucasian Youth

**DOI:** 10.3390/ijerph18094667

**Published:** 2021-04-27

**Authors:** Meizi Wang, Jianhua Ying, Ukadike Chris Ugbolue, Duncan S. Buchan, Yaodong Gu, Julien S. Baker

**Affiliations:** 1 Faculty of Sports Science, Ningbo University, Ningbo 315211, China; nbuwangmeizi@aliyun.com; 2 College of Science Technology, Ningbo University, Ningbo 315211, China; yingjianhua@nbu.edu.cn; 3 Institute for Clinical Exercise and Health Sciences, School of Science, University of the West of Scotland, Hamilton, Scotland ML3 0JB, UK; U.Ugbolue@uws.ac.uk (U.C.U.); Duncan.Buchan@uws.ac.uk (D.S.B.); 4 Department of Sport and Physical Education, Hong Kong Baptist University, Hong Kong 999077, China

**Keywords:** ethnicity, South Asians, adiposity, cardio metabolic risk, obesity

## Abstract

(1) Background: Scotland has one of the highest rates of obesity in the Western World, it is well established that poor weight profiles, and particularly abdominal obesity, is strongly associated with Type II diabetes and cardiovascular diseases. Whether these associations are apparent in ethnic population groups in Scotland is unclear. The purpose of this study was to examine the associations between different measures of fatness with clustered cardio metabolic risk factors between Scottish South Asian adolescents and Scottish Caucasian adolescents; (2) Methods: A sample of 208 Caucasian adolescents and 52 South Asian adolescents participated in this study. Stature, waist circumference, body mass index, blood pressure, physical activity, and cardiovascular disease (CVD) risk were measured; (3) Results: Significant, partial correlations in the South Asian cohort between body mass index (BMI) and individual risk factors were generally moderate. However, correlations between Waist circumference (WC) and individual risk factors were significant and strong. In the Caucasian cohort, a significant yet weak correlation between WC and total cholesterol (TG) was noted although no other associations were evident for either WC or BMI. Multiple regression analysis revealed that both BMI and WC were positively associated with CCR (*p* < 0.01) in the South Asian group and with the additional adjustment of either WC or BMI, the independent associations with clustered cardio-metabolic risk (CCR) remained significant (*p* < 0.005); (4) Conclusions: No positive relationships were found between BMI, WC, and CCR in the Caucasian group. Strong and significant associations between measures of fatness and metabolic risk were evident in Scottish South Asian adolescents.

## 1. Introduction

The global pandemic of diabetes, obesity, and cardiovascular disease (CVD) continues to rise at an alarming rate [1]. Approximately 23% of the CVD burden and 44% of the diabetes burden is attributed to overweight and obesity, which itself is the fifth leading cause of global death [1,2]. By 2030, it is expected that diabetes will become the seventh leading cause of death, with half a billion people being diagnosed with the disease [2]. Scotland has one of the highest rates of obesity in the Western World with 39.5% of men and 32.2% of women classified as overweight with a further 26.6% of men and 28.8% of women classified as obese [3,4]. The number of individuals with Type II diabetes in Scotland is increasing by 4% annually. This is equivalent to 13,000 individuals every year [2,5].

It is well established that poor weight profiles, and particularly abdominal obesity, is strongly associated with Type II diabetes and CVD [5,6,7,8]. Obesity-related risks are graded according to body mass index (BMI) and waist circumference (WC). Studies have shown that BMI and WC independently contribute to the efficient prediction of abdominal subcutaneous, nonabdominal, and visceral fat in a large group of white men and women with varying ages and adiposity [9]. In addition, research has indicated that the involvement of visceral adiposity has a greater impact on cardiovascular disease when compared with general fat mass, especially in subjects with a high BMI of more than 25 kg/m2 [10,11]. Much of the information reported is based on studies involving Caucasian adult populations. Whether these associations are apparent in ethnic population groups remains unclear. However, some evidence does suggest that South Asians are at a greater risk of developing CVD and adverse health risk factors at lower and similar degrees of abdominal obesity.

It has been documented that South Asians living in the UK are up to six times more likely to develop Type II diabetes than their white indigenous counterparts. In 2004, it was estimated that children of South Asian origin were 13 times more likely to develop Type II diabetes. It has also been reported that children of South Asian origin are significantly less physically active than Caucasian British children [12,13]. Nevertheless, the sparse research studies completed in British South Asian cohorts are derived from predominantly English South Asian groups. There has been little or no research data from the other three UK nations, particularly Scotland, where the notoriously poor diet and physical activity levels could have further negative implications for health profiles [12].

Although the mechanisms for the increased level of risk in South Asians remain unclear, dietary and physical activity behaviors, genetics, cultural, and environmental factors have all been implicated as key contributors [12]. Individual risk factors, and more recently clustering of risk factors, along with poor behavioral habits are known to track moderately from adolescence into adulthood. This process creates a long-term exposure to undesirable factors known to significantly increase future risk of CVD [14,15,16,17]. Despite this, no studies to date have examined the different associations between Scottish South Asians and Caucasians. The purpose of this study, therefore, was to examine if the associations between different measures of fatness with clustered cardio-metabolic risk factors are different between Scottish South Asian adolescents and Scottish Caucasian adolescents.

## 2. Materials and Methods

### 2.1. Subjects

A group of 52 Scottish South Asian adolescents aged 14–18 years old (22 males and 30 females) and 208 Scottish Caucasian adolescents with ages ranging between 16 and 18 years old (70 females and 138 males, 17 ± 1 years of age) participated in the study, with data collection completed in 2019. The South Asian adolescents studied in this experiment were difficult to recruit. As a result, South Asian Participants were recruited in conjunction with the Scottish ethnic minority sports association (SEMSA) database of ethnic minority adolescents in Glasgow. The Caucasian cohort was recruited from a South Lanarkshire school in the West of Scotland. This experimental protocol was approved by the University of the West of Scotland ethics committee (number #4-8-15-001). All subjects were medically screened prior to testing. Subjects with a prior history of hyperlipidemia, hypertension, diabetes, cardiomyopathy, or coronary heart disease were excluded from the study, as well as physically and mentally disabled subjects. Participants were required to maintain their daily routines throughout the study and not to change their lifestyle and dietary habits. 

### 2.2. Anthropometric and Physiological Measures

A calibrated electronic weighing scale (Seca 880, Digital Scales, Seca Ltd., Birmingham, UK) was used to measure body mass in normal daily clothing without shoes to the nearest 0.1 kg. Stature without shoes was measured to the nearest 1 mm (Seca Stadiometer, Seca Ltd., Birmingham, UK). Body mass index (BMI) was calculated using standard equations ((body mass (kg)/(height (m)^2^), Waist circumference (WC) was measured at the midpoint between the lower ribs and iliac crest following standard procedures. Weight status for both cohorts was defined using the criteria proposed by Cole et al., 2000 [18]. Blood pressure was recorded following 10 min of rest in a seated position. Systolic blood pressure (SBP) and diastolic blood pressure (DBP) was measured with an automated BP monitor (Omron Healthcare UK Ltd., Milton Keynes, UK). The cuff was placed tightly on the upper left arm with the participants while comfortably seated. In total three measurements were carried at 1 min intervals. The second and third results were used in the analysis. Subjects’ sexual maturity was evaluated using Tanner’ s 5-stage scale for girls’ and boys’ [19]. Physical activity level was estimated using the validated physical activity questionnaire for adolescents (PAQ-A) with a score ranging from 0 to 5, with 0 representing no physical activity, and 5 reflecting a high level. 

### 2.3. Metabolic Measurements

During the time period between 9:00 a.m. and 11:00 a.m., overnight fasted blood samples were collected by qualified phlebotomists, experienced in pediatric sampling techniques. Times were standardized to minimize the effects of diurnal variation. Participants were instructed to sit and rest for at least 30 min prior to blood sampling to control for plasma volume shifts. All blood samples were analyzed for insulin, glucose, adiponectin, total cholesterol (TC), triglycerides (TG), and high-density lipoprotein (HDL-C).

Blood analysis was performed using standard procedures. TG and TC were measured using enzymatic methods (Randox, Antrim, UK) and a Camspec M107 spectrophotometer (Camspec, Leeds, UK). Concentration of HDL-C was determined after precipitation of very low density and low-density lipoproteins by the addition of phosphotungstic acid in the presence of magnesium ions. Glucose was measured using the glucose oxidase method (Randox, Antrim, UK) and analyzed using a Camspec M107 spectrophotometer (Camspec, Leeds, UK). Plasma insulin was analyzed using a Camspec M107 spectrophotometer and easily available immunoassay kits (ALPCO, Salem, NH, USA). Homeostatic assessment model (HOMA) was calculated (product of fasting glucose and insulin divided by the constant of 22.5) to provide an indication of insulin resistance. Concentration of TG was calculated with a specific ELISA kit (R&D Systems, Abingdon, UK) and an MRX microplate reader (Dynatech laboratories, Chantilly, MA, USA).

### 2.4. Clustered Cardio Metabolic Risk (CCR)

A continuous risk score representative of a clustered cardio-metabolic risk (CCR) was calculated from the following variables: Systolic blood pressure (SBP), HOMA, TG, and TC:HDL ratio. These variables were included as each individual score is a well-established independent CVD risk factor. The z-scores of the individual risk factors were summed to create the CCR score. A lower CCR score recorded was associated with a healthier profile.

### 2.5. Statistical Analysis

All data are presented as mean ± SD and were analyzed using IBM SPSS 22.0 (SPSS, Inc., Chicago, IL, USA) statistics with values of *p* ≤ 0.05 considered to be statistically significant throughout. All variables were checked for normality of distribution before the analysis, and transformations were applied when necessary. HOMA score, TG, and TC/HDL-C were normalized by natural logarithmic transformation. 

Levene’s test was used to assess the assumption of homogeneity of variance; for variables where the assumptions of normality and/or homogeneity of variance were invalid, the non-parametric Mann–Whitney test was used. When assumptions of normality and/or homogeneity of variance were valid independent t-tests were performed to analyze differences between the South Asian and Caucasian cohorts. As no significant interaction was found for age, maturation, and gender in each cohort, all analyses were performed with boys and girls together to increase statistical power. Partial correlations adjusted for Gender, Age, Maturation, and Physical Activity levels were used to examine bivariate correlations of BMI and WC with individual CVD risk factors and the CCR score. 

Two separate multiple regression models were used to examine the associations of BMI and WC with the clustered cardio-metabolic risk score. Model 1 included BMI or WC and was adjusted for age, gender, pubertal stage, and physical activity. In model 2, additional adjustments were made for the other predictor variables to test independent associations

## 3. Results

Descriptive results for all variables are reported in Table 1. The South Asian Group were significantly shorter and lighter, with a significantly lower WC, TG level, and TC: HDL ratio and a significantly greater SBP than the Caucasian group (*p* < 0.05) (Table 1). No significant differences were reported for PA, BMI, Insulin, Glucose, and HOMA. A total of 21.84% of the Caucasian population was overweight, whereas only 12.5% of South Asians were overweight. However, 6.25% of the South Asian population were obese compared to only 0.97% in the Caucasian population (Table 1). Partial correlations were moderate to strong and significant for all variables apart from BMI and TG in the South Asian cohort (Table 2). WC was significantly correlated with all CVD risk factors apart from with SBP (Table 2). Few correlations were identified in the Caucasian group and included WC and BMI, and a weak correlation between WC and TG as expected (Table 3).

Regression analysis revealed that both BMI and WC were positively associated with CCR (*p* < 0.01) after adjustment for age, sex, maturation status, and physical activity in the South Asian group (Table 4). In the BMI model additional adjustment for WC strengthened the association (*p* < 0.001), whereas in the WC model additional adjustment for BMI weakened the relationship but remained significant (*p* < 0.005). No positive relationships were reported in the Caucasian group (Table 5).

## 4. Discussion

This study examined whether the associations between different measures of fatness (WC and BMI) with clustered cardio-metabolic risk differed between Scottish South Asian adolescents and Scottish Caucasian adolescents. Findings revealed a strong and positive relationship between different measures of fatness, CVD and Type II diabetes risk factors. WC emerged as the stronger independent factor in comparison to BMI in the South Asian cohort, which is consistent with other findings [20,21,22,23]. Our results agree with previous research that illustrates the strong association between central adiposity and a poor cardio-metabolic profile [21,24,25,26]. The South Asian cohort had a significantly lower WC and similar BMI (Table 1); however, both WC and BMI strongly correlated with individual CVD risk factors and the CCR, while the Caucasian cohort showed no such associations (Table 2 and Table 3). Additionally, the multiple linear regression analysis revealed a significant association between WC and CCR, and BMI with CCR, in the South Asian cohort, although no such relationship was evident in the Caucasian group (Table 4 and Table 5). Furthermore, multiple linear regressions revealed that although both BMI and WC are powerful indicators of CCR, WC appeared to be more strongly associated with CCR than BMI in the South Asian group. In addition, the prevalence of overweight was greater in the Caucasian group (Table 1) even though no significant associations between BMI, WC, and CVD risk factors were noted. 

The World Health Organization (WHO) concluded previously that existing BMI cut-off points are not ethnic specific leading to several false negatives [22,23] in the assessment of BMI. South Asians have a significantly greater proportion of individuals with cardio-metabolic risk factors below the current cut-off points while having a higher percentage body fat than Europeans at a given BMI [22,24,27,28]. Studies by Whincup et al. 2010 [29], confirm the findings of the WHO panel by reporting adolescents of South Asian origin to have a greater CVD risk profile when compared to their Caucasian counterparts at lower BMI levels. This also suggests that the current primary diagnostic tools for assessing CVD risk are not ethnically viable [22,28,29,30,31]. The results from this study confirm the outcome of the WHO panel and the studies of Whincup et al. 2010 [26]. Although there were a greater percentage of overweight individuals in the Caucasian group (Table 1) there were no significant correlations between WC, BMI, and any CVD risk factors (Table 3 and Table 5). This clearly indicates that current cut-off points are not suitable for determining weight status in a Scottish South Asian population. Our results illustrate a strong relationship between measures of fatness and CVD risk factors with an apparent lower prevalence rate of overweight in this group. As a primary diagnostic tool current cut-off points are inaccurate for the South Asian population with the incorrect determination of weight status not allowing for early detection and placement of preventative measures. Ethnic differences in cardio-metabolic risk profiles have been widely reported in both Adults and adolescents and in some studies in children as young as six years of age [20,22,23,24,25,27,31]. Generally, these investigations have noted that risk profiles tended to be higher in South Asian populations when compared to Caucasian populations. However, no such comparisons to date have been made in a Scottish population, with most of the work being based in European countries and England where it is reported that the child and adolescent population has more favorable physical activity and dietary behavior profile compared to their Scottish Caucasian counterparts [1,4,13,32,33,34,35]. National surveys have revealed the intake of fruit and vegetables to be significantly lower in Scotland while the prevalence of adverse behaviors, such as smoking and alcohol consumption to be greater [33]. This may help to provide reasons for the differences observed between the groups. 

The strong correlations between WC, BMI, and the individual risk factors along with the CCR in the South Asian Cohort appears to be more significant and pronounced than in the Caucasian group. The mechanism in which this relationship exits has been a focal point in recent years with studies reporting South Asians to have a great percentage of body fat at a given BMI and more importantly visceral adipose tissue [36,37]. Visceral fat is thought to raise circulating levels of free fatty acids, stimulating increased secretion of Apo-b lipoproteins and impairing insulin sensitivity leading to raised blood glucose levels [27,29,30,36,37].

Genetic and environmental factors have also been proposed to increase the accumulation of visceral adipose tissue in South Asians when compared to Caucasians [21,24,25,26]. Physical activity levels in both groups were not high and a large majority of youth from both groups did not meet the recommended physical activity guidelines [34]. According to the results of the PAQ-A, physical activity levels for the South Asians and Caucasians were 1.5–2.9 respectively. This lack of physical activity may contribute to an increased genetic influence in the increased accumulation of visceral fat. The low physical activity levels would be compromised further by poor quality Scottish diets high in saturated fats leading to a double burden of poor environmental factors, and predisposed genetic influences [26,29,38,39,40]. Early detection of a poor health profile is therefore of paramount importance [41] and continued research in this high-risk group would allow for the development of ethnic specific diagnostic tools, with amendments to current BMI cut off points as previously suggested by others [22]. This would allow practitioners to identify at risk individuals at an earlier stage and implement preventative measures with the intended outcome of reducing this growing epidemic in South Asian groups. There are some limitations in this research study. Firstly, the significantly smaller sample size for the South Asian population compared to the Caucasian group may have influenced the findings. It was very difficult to recruit South Asian adolescents for this experimental research project due to their cultural, social, and religious beliefs. The number recruited here, although relatively small, has made an important and significant contribution to the research topic, and without cooperation from this group these research findings would not have been possible. However, further large-scale studies are needed with larger sample sizes to confirm the findings of this study. It would have been impossible to have recruited the subjects for this study without the help of the Scottish ethnic minority sports association (SEMSA). Secondly, cardiorespiratory fitness was not assessed. Some studies have noted that examining the prevalence of CVD risk factors may not only be related to obesity or physical activity but also to cardiorespiratory fitness in a youth population [42,43]. Furthermore, this study may have benefited from additional data sets, including information relating to social background and behavioral variables that may have informed the outcome measures observed.

## 5. Conclusions

This study did not find any significant differences in overall cardio-metabolic risk score between the two groups. However, we did report strong and significant correlations between BMI, WC, and all individual CVD risk factors in the South Asian Cohort but not in the Caucasian cohort. This clearly suggests a strong relationship between measures of fatness and CCR. The future use of both BMI and WC as tools for assessing CCR in South Asians with the view of lowering cut-points for the South Asian population needs serious consideration.

## Figures and Tables

**Table 1:** Characteristics of the study population.

Variables (Units)	Total (*n* = 256)	South Asian (*n* = 52)	Caucasian (*n* = 208)	*p*-Value
Age (year)	16.7 ± 0.8	16.4 ± 1.4	16.7 ± 0.6	0.07
Maturation	4 ± 1	5 ± 0.5	4 ± 1	0.00
Physical Activity	2.2 ± 0.7	2.2 ± 0.7	2.2 ± 0.7	0.742
Height (m)	1.7 ± 0.1 (254)	1.65 ± 0.1	1.7 ± 0.1 (206)	0.00
Body Mass (kg)	64.2 ± 10.4 (254)	59.7 ± 12	65.2 ± 9.8 (206)	0.001
BMI (kg/m^2^)	22 ± 3 (254)	21.6 ± 3.7	22.1 ± 2.9 (206)	0.231
Waist Circumference (cm)	74.2 ± 8.4 (253)	72.1 ± 13.4 (45)	74.6 ± 6.8	0.021
Weight Status (%)				
Healthy	78	81	77	-
Overweight	20	13	22	-
Obese	2	6	1	-
SBP (mm/HG)	121 ± 13.8 (250)	125 ± 13.5 (47)	120 13.8 (203)	0.027
Insulin (Iu/mL)	7.2 ± 4.9 (237)	9.4 ± 9 (39)	6.7 ± 3.5 (198)	0.802
Glucose (mMol/L)	4.8 ± 1.1 (246)	4.9 ± 1.1 (47)	4.7 ± 1.2 (199)	0.369
HOMA	1.5 ± 1.1 (230)	2 ± 1.8 ((39)	1.4 ± 0.8 (191)	0.482
Trigs (mMol/L)	1 ± 0.4 (253)	0.8 ± 0.5 (47)	1 ± 0.4 (206)	0.00
TC:HDL Ratio	2.6 ± 1.2 (248)	2.2 ± 0.9 (47)	2.7 ± 1.2 (201)	0.005

BMI, body mass index; HOMA, homeostasis model assessment; SBP, systolic blood pressure; TC: HDL Ratio, total cholesterol and High-Density Lipoprotein Ratio; TG, triglycerides. Data are presented as mean ± standard deviation, except for weight status (%). Differences between South Asians and Caucasians were examined by independent t-tests, except for Age, Maturation, Weight, BMI, Waist Circumference, Insulin and HOMA (Mann–Whitney U Test). Where *n* ≠ denoted number, actual sample number is presented in parenthesis.

**Table 2:** South Asian partial correlations (adjusted for Gender, Age, Maturation, and Physical Activity levels) for individual CVD risk factors and clustered cardiometabolic risk with BMI and WC.

	SBP	HOMA	TG	TC: HDL	CCR
BMI	0.378 *	0.409 **	0.306	0.38 *	0.579 ***
WC	0.355 *	0.464 **	0.393 *	0.555 ***	0.700 ***

CCR, clustered cardiometabolic risk; BMI, body-mass-index; WC, waist circumference; SBP, systolic blood pressure; HOMA, homeostasis assessment model; TG, triglycerides; TC: HDL, total cholesterol and high-density lipoprotein ratio. * *p* < 0.05; ** *p* < 0.01; *** *p* < 0.001.

**Table 3:** Caucasian partial correlations (adjusted for Gender, Age, Maturation, and Physical Activity levels) for individual CVD risk factors and clustered cardio-metabolic risk with BMI and WC.

	SBP	HOMA	TG	TC:HDL	CCR
BMI	0.082	0.017	0.125	0.066	0.129
WC	0.05	0.043	0.177 *	0.28	0.131

CCR, clustered cardio-metabolic risk; BMI, body-mass-index; WC, waist circumference; SBP, systolic blood pressure; HOMA, homeostasis assessment model; TG, triglycerides; TC: HDL, total cholesterol and high density lipoprotein ratio. * *p* < 0.05.

**Table 4:** Standardized regression coefficients examining the association of BMI and WC with clustered metabolic risk in the South Asian.

	Body Mass Index				Waist Circumference			
	R^2^	β	R^2^ Change	*p*-Value	R^2^	β	R^2^ Change	*p*-Value
Model 1	0.359	0.372	0.359	0.005	0.41	0.455	0.41	0.001
Model 2	0.514	0.455	0.155	0.001	0.514	0.372	0.104	0.005

Model 1 is adjusted for adjusted for Gender, Age, Maturation and Physical Activity levels. Model 2 is adjusted for all covariates plus Body Mass Index or Waist Circumference.

**Table 5:** Standardized regression coefficients examining the association of BMI and WC with clustered metabolic risk in the Caucasian Cohort.

	Body Mass Index				Waist Circumference			
	R^2^	β	R^2^ Change	*p*-Value	R^2^	β	R^2^ Change	*p*-Value
Model 1	0.024	0.116	0.024	0.202	0.018	0.06	0.018	0.509
Model 2	0.026	0.06	0.002	0.509	0.026	0.116	0.008	0.202

Model 1 is adjusted for adjusted for Gender, Age, Maturation, and Physical Activity levels. Model 2 is adjusted for all covariates plus Body mass Index or Waist circumference.
